# Traditional and Complementary Medicine in Tanzania: Regulation Awareness, Adherence and Challenges

**DOI:** 10.34172/ijhpm.2021.51

**Published:** 2021-06-09

**Authors:** Phares Gamba Mujinja, Happiness Pius Saronga

**Affiliations:** Department of Behavioural Sciences, School of Public Health and Social Sciences, Muhimbili University of Health and Allied Sciences, Dar es Salaam, Tanzania.

**Keywords:** Traditional Medicine, Complementary Medicine, Regulation, Awareness, Adherence, Tanzania

## Abstract

**Background:** The demand for and use of Traditional and Complementary Medicine (T&CM) has recently increased worldwide drawing a public health attention including malpractice, which puts the health of its clients at risk. Despite efforts made by Tanzania to integrate T&CM in the health system to protect the clients, regulating the subsector has remained a challenge due to lack of information and operational factors facing the regulatory frameworks in Tanzania. The aim of this study was to determine the extent of imperfect information, regulation adherence and challenges among T&CM practitioners and regulators in Tanzania.

**Methods:** In-depth interviews were carried out with T&CM practitioners in Dar es Salaam Region in Tanzania, and officials from the Ministry of Health and the study municipals. Purposive and snowballing approaches were used to select study participants. Thematic data analysis was done with the help of NVIVO.

**Results:** Awareness of regulations and tools used for regulating the T&CM operations among practitioners was generally very low. There was fragmentation of knowledge on what they were practicing as well as on awareness of the regulations, and what is regulated. Practitioners argued that they cannot be controlled by conventional medical trained personnel. Regulators at municipal level reported to have had no knowledge, interest, and time to work on T&CM. Lack of adequately trained and qualified manpower, lack of financial resources, poor transport and other infrastructure at the municipal regulatory units aggravated non-adherence to regulations, and therefore rendered ineffectiveness to the regulatory framework.

**Conclusion:** Existence of imperfect information on T&CM among regulators and practitioners affect effectiveness of T&CM regulatory process. Awareness of regulations among practitioners, presence of knowledgeable regulators, as well as capacity would facilitate adherence to regulations.

## Background

**Key Messages**

**Implications for policy makers**

- There is existence of imperfect information among practitioners and regulators in Traditional and Complementary Medicine (T&CM) industry, and this affects the effectiveness of T&CM regulatory process.
- Lack of adequately trained and qualified manpower, lack of financial resources and poor infrastructure aggravates non-adherence to T&CM regulations.
- It is critical to generate awareness and knowledge among T&CM practitioners and regulators, as well as address human, financial and infrastructural challenges to facilitate adherence to T&CM regulations.

**Implications for the public**

This research shows existence of imperfect information among Traditional and Complementary Medicine (T&CM) practitioners and regulators. In this case both practitioners and regulators do not have adequate information on T&CM affecting regulation of T&CM and risking public health safety. Other challenges affecting the regulatory process include lack of trained personnel, financial constraints, and poor infrastructure. This research gives recommendations on addressing the regulatory challenges to ensure T&CM consumers are protected from health risks that may arise from unregulated T&CM practice.

Recently, the demand for and use of traditional and complementary medicine (T&CM) has increased in both developed and developing countries.^1-3^ T&CM merges the terms traditional medicine and complementary medicine, encompassing products, practices, and practitioners.^3^ Traditional medicine is the sum of the knowledge, skills and practices based on theories, beliefs, and experiences indigenous to different cultures, whether explicable or not, used in the maintenance of health as well as in the prevention, diagnosis, improvement, or treatment of physical and mental illness. Complementary medicine is a broad set of healthcare practices that are not part of that country’s own traditional or conventional medicine and are not fully integrated into the dominant healthcare system. In this study we concentrate on T&CM practice. The T&CM practice has been a public health concern because it is also accompanied with malpractice that puts the health of its clients at risk. The malpractices include false labeling and advertising of T&CM products; false claims of ‘magic’ and efficacious treatment; and false self-proclaiming of ‘doctor’ title by practitioners.^4,5^

 Malpractice in T&CM may be facilitated by presence imperfect information, among other factors. Imperfect information exists when either the practitioners or the regulators do not have full knowledge about the T&CM practice, regulations, and what is being regulated. In this study imperfect information refers to lack of accessibility, awareness, and clarity of available T&CM information among T&CM regulators and practitioners. Presence of imperfect information among practitioners and regulators can easily cause non-adherence to and poor implementation of regulations, respectively. If access to T&CM information is limited then possibly practitioners may not be able to adhere to some regulations and regulators may not be able to regulate effectively.

 Information on T&CM practice is critical for public health safety because many people in developing countries access T&CM when in need of healthcare before accessing contemporary medicine or in conjunction with contemporary medicine.^6-10^ This makes accessibility to information on T&CM regulations among practitioners and regulators critical to public health because regulations protect T&CM consumers against malpractice.^11^

 Imperfect information is more pronounced in T&CM practice due to multiple groups of practitioners practicing distinctive modes of the profession.^12^ In most developed countries, the fragmentation among practitioners is due to each group’s specialized training in T&CM branches like acupuncture, herbal medicine, homeopathy, Ayurveda, chiropractic, osteopathy, and naturopathy.^13^ On the contrary, in most developing countries the fragmentation is mostly due to intra-practitioner’s knowledge differences and origin of the practice; also, some have no formal specialized training in what they practice.^14^

 The professional knowledge heterogeneity within practitioner groups in developed countries has resulted into demand for self-regulatory framework among different specialized groups. While others regard T&CM as nutritional supplements, hence not strictly regulated as medicines, others force them into the conventional medicine regulatory system or disregard them.^13,15^ In addition, the intra-practitioner knowledge differences in developing countries might have contributed to aggravation of the differences in awareness, and consequently, adherence to regulations.^13^

 Furthermore, the existence of imperfect information in T&CM practice calls for governmental regulatory action to protect customers against risky practices. The World Health Organization (WHO) developed a Traditional Medicine strategy (2014-2023) to guide T&CM policies, plans and regulatory process. It emphasizes, among other things, that for an effective regulatory framework: there must be clear and adequate legislation, regulation, guidelines and procedures; human and financial resources; transparency, cooperation and collaboration between regulating authority and other stakeholders including the practitioners.^2^

 Therefore, in order to reduce consumer risk, improve safety and quality standards, and improve awareness and adherence to regulations, some countries have integrated T&CM in the health system in various ways.^1^ In Tanzania, the Traditional Medicine Act was enacted in 2002 and a Traditional Medicine unit in the Ministry of Health was initiated.^16^ However, studies show that despite the integration of T&CM in the health system, the regulatory authorities and their operations are still ineffective and less efficient.^2^ Studies have found a knowledge gap on T&CM operations even amongst regulators.^8^

 The aim of this study was to determine the extent of imperfect information, regulation adherence and challenges among T&CM practitioners and regulators in Tanzania. Past studies on regulation of T&CM have focused more on safety of products, standards and quality of herbal medicines.^13-15,17^

## Methods

###  Study Design

 We employed a cross-sectional explorative design to conduct the study in Dar es Salaam region, the largest city and business capital of Tanzania. The site was purposely chosen due to its large number of T&CM premises. A qualitative approach was used, where a series of key informant interviews were carried out with T&CM practitioners and regulators.

 We opted for the in-depth key informant interview approach because we aimed to obtain respondents’ opinions, feelings, perceptions as well as awareness on T&CM regulatory process.^18,19^

###  Study Population 

 The study population included three major players in the regulatory process of the T&CM industry: regulators from the Ministry of Health; regulators from three Dar es Salaam Municipals; and T&CM practitioners. Regulators roles include implementation of regulatory guidelines, provision of regulatory information to practitioners, licensure, and inspection.

###  Sample Size and Sample Selection

 We aimed at obtaining different views and perspectives from diverse practitioners and regulators in the T&CM practice, therefore we used purposive and snowballing sampling techniques to obtain respondents. We obtained a list of all registered T&CM premises from the Traditional Medicine regulatory board, from which we conveniently selected the study participants. Practitioners included sellers of locally manufactured products and sellers of products from other sources (premises that sold products prepared by owners of premises, products prepared by other practitioners and products imported from other countries). We purposefully selected 15 premises, 5 from each municipal council, and interviewed one person from each T&CM premises. Respondents were asked to identify other practitioners who could contribute information to the study. Using snowballing technique, 5 more practitioners were identified and subsequently added to the list of participants and interviewed.

 Regulators included two officials from the Ministry of Health and three officials from three Municipals in Dar es Salaam.

###  Data Collection Instruments 

 Interview guides for the two respondent groups were developed and used for data collection, one for practitioners, and one for regulators including municipal inspectors, and Ministry of Health officials. The interview guides were designed to capture regulators and practitioners opinions, feelings, and perspectives on T&CM regulatory framework. The practitioners guide was specifically used to explore awareness of regulations and adherence to regulations (Box 1). The questions inquired on awareness of legislation, decrees and regulations, sources of information, licensing procedure, knowledge on tools used to regulate, supervisory visits by regulators as well as the supervisory process, regulatory capacity, extent of adherence to regulations, and problems/challenges related to adherence.

**Box 1. In-Depth Interview Guide for Practitioners and Regulators on T&CM Regulation Awareness, Adherence, and Challenges**

**Practitioner Question Guide**

1. What do you understand by being regulated? (Probe on awareness of T&CM legislations, decrees and regulations, sources of information)
2. Can you mention what the regulations require you to do? (Probe on licencing/registration process)
3. Have you ever been regulated? (Probe on what/? how frequent?)
4. What are the instruments that are used to regulate?
5. Do you understand the content of these regulatory instruments?
6. In your opinion are these instruments effective in regulating the sector?
7. Have you ever been invited to attend a meeting by any of the regulatory bodies and what did you learn there? (Probe on how many times in the past year?)
8. In the past one year, how many times were you visited by regulators? What did they do?
9. Do you think the regulators have the capacity of doing their work?
10. Are the regulators acting on what they are supposed to do?
11. In your opinion do you think regulations address the most important issues?
12. How do you rate the capacity of the regulatory bodies in regulating the subsector?
13. Do you adhere to the regulations?
14. What factors influence your adherence to the regulations?
15. In your opinion what are the challenges affecting the regulatory process?
16. What would you suggest to improve the regulatory process?

**Regulator Question Guide**

1. What do you understand about the regulatory process? (Probe on awareness of legislation, decrees, and regulations)
2. What are the instruments that are used to regulate? (Probe on licencing, penalties, and legal enforcement)
3. Do you understand the content of these regulatory instruments?
4. In your opinion are these instruments effective in regulating the sector?
5. How many consultative meetings have you had with practitioners? Which practitioners?
6. How often do you visit practitioners?
7. What do you normally inspect when you visit?
8. What are the things that you normally regulate?
9. Do you give guidance materials to practitioners? (Probe on the main content of these guidance materials, and whether they conduct seminars to elaborate on the guidance materials)
10. How do you usually communicate with practitioners?
11. Do you have any feedback mechanism to and from practitioners? How often?
12. How do you rate the capacity of the regulatory bodies in regulating the subsector? Why?
13. In your opinion what are the challenges affecting the regulatory process?
14. What would you suggest to improve the regulatory process?

-------------

Abbreviation: T&CM, traditional and complementary medicine.

The regulators’ guide broadly consisted of questions related to regulatory process, legislation, decrees and regulations, regulatory tools, regulatory capacity, supervision and inspection, information sharing and collaboration with practitioners, and operational challenges faced by the regulatory authorities. Interview guides were pre-tested in Dar es Salaam, in clinics and traditional shops that were not included in the study to ensure reliability and trustworthiness. Interview questions were revised according to the pre-test results.

###  Data Collection Procedure

 Before approaching the regulators for interviews, we sought permission from District Medical Officers who are in charge of the health sector in the municipals. All respondents were approached and informed of the objectives of the study; signed a consent form, which included participation rights, study confidentiality, and approval to be tape-recorded during the interview; and were invited to a face to face interview. The process of interviewing continued until saturation point was reached, until no new information was being obtained from participants, and the list of respondents identified through snowballing was exhausted.^18,19^

 Interviews were conducted by 2 researchers. The first researcher is a male university Professor with a PhD in public health, and over 20 years’ experience in qualitative research. The second researcher was a male (now deceased) with an MPH degree, and over 5 years’ experience in qualitative research. Both researchers were thoroughly trained on research objectives and data collection procedure. The researchers had no direct relationship with the study participants; interaction between participants and researchers was objective and followed all ethical guidelines. Interviews with practitioners were conducted at practitioners’ premises and interviews with regulators were conducted at regulators’ offices. Privacy during interviews was observed, non-participants were not allowed in the interview area. Repeat interviews were not conducted. Each interview lasted for about 35 to 45 minutes (average 40 minutes). All interviews were conducted in Kiswahili language. Along with tape recording, the interviewers took field notes to facilitate data analysis, and as a backup in case certain data elements were missed during taping of interviews. All interviews were conducted in June 2017.

###  Data Analysis

 All interviews were transcribed verbatim and thematic analysis was applied in data analysis. We started data analysis by carefully reading the narratives.^18,19^ Data were arranged as themes emerged from the interview narratives. Recurring issues and patterns from the data were detected through coding of respondents’ narratives and not through predetermined thematic codes.^18^ NVivo software version 11 was used for data management.

 Thorough reading and re-reading of each transcript from all sources was done. The first step of reading of the scripts was done to gain an initial general insight of the text, as well as relate them to the research objectives. In the second step, each question in the interview guides was placed under a respective objective and categorized as a general theme, while the subsequent questions were treated as subthemes. The responses were classified under the respective themes and subthemes. Statements that did not fit under any of the research questions were regarded as miscellaneous, and therefore not used.

 Themes and subthemes were linked to the research questions, and subsequently used to describe the findings.

 Data from the three sources (transcripts, audio, and field notes) were revisited several times to verify and confirm the themes, subthemes and patterns that were identified and coded. After the primary coding of all transcripts, analysis sheets were independently re-coded into specific themes and sub-themes by the principal researcher and a co-investigator. Any coding disagreements between the two were resolved by other qualitative analysis experts in a series of discussions until a final list of themes and sub-themes was generated. We finally agreed on the number of general themes and subthemes.

## Results

 A total of 17 practitioners and 5 regulators accepted to participate in this study. Study participants constituted 3 municipal T&CM inspectors (1 female and 2 male participants), 2 female participants from Directorate of Traditional and Alternatives in Ministry of Health, and 17 practitioners from T&CM clinics, outlets and facilities (6 females and 11 males). A total of 22 respondents (practitioners and regulators) were interviewed. Respondents’ ages ranged between 20-56 years.

 Three practitioners had been practicing for a period of 1 to 10 years, 11 practitioners had been practicing for a period of 11 to 20 years, and 3 practitioners had above 21 years of experience (Table 1). Practitioners were practice- and/or product-based because we recruited participants from premises that practiced T&CM and/or sold products.

 Regulators were involved with implementation of regulatory guidelines, provision of regulatory information to practitioners, licensure, and inspection.

**Table 1:** T&CM Practitioners’ Experience

**Experience Years**	**Frequency **
1 to 10	3
11 to 20	11
21 and above	3
Total	17

Abbreviation: T&CM, traditional and complementary medicine.

 During data analysis 3 main themes were generated; awareness of T&CM regulatory framework, adherence to regulations, and barriers and challenges of the regulatory process (Table 2). Four subthemes on awareness of T&CM regulatory framework were awareness of and attitude towards existence of regulatory bodies and regulations; awareness of regulatory instruments and enforcements; supervision, inspection and information sharing; and regulators’ perspective on supervision and sharing of information. Two subthemes on adherence to regulations were financial cost and complexity of laws and regulations. One subtheme on barriers and challenges of the regulatory process include capacity, knowledge, interest, and communication. All elements of the themes and subthemes are presented and discussed in the subsections below.

**Table 2:** Thematic Analysis Coding Illustration

**Main Theme**	**Sub-theme**	**Quote**
Awareness of T&CM regulatory framework	Awareness of and attitude towards existence of regulatory bodies and regulations	*“I have been practicing traditional/alternative medicine for twelve years I have heard nothing called regulations. I wonder who set them and who was involved in developing them. Do they think this is conventional medicine?”* (Participant 2).
	Awareness of regulatory instruments and enforcements	*“I know nothing about the instruments and their contents that regulators use to regulate our premises and practice” *(Participant 9).
	Supervision, inspection, and information sharing	*“No regulator has ever visited this premises ever since I started practicing traditional medicine for the past twelve years now”* (Participant 3).
	Regulators’ perspective on supervision and sharing of information	*“…because I have poor knowledge in this field … I am not sure if I can confidently share such information with practitioners…they may be more informed than me”* (Participant 18).
Adherence to regulations	Financial cost	*“One of the things that I remember in the regulatory guidelines is the structure and content of the premise, to adhere to this; it means a lot of money which most of us cannot afford to construct the premises the way it is needed by the guideline. … it is very costly to register one herbal product; it costs around TZS 800 000 to TZS 1 000 000 (equivalent to $380 to $476). Cost of renting a standard premise as required by regulation one needs a lot of money”* (Participant 9).
	Complexity of laws and regulations	*“…it is a barrier to the practice…you have to travel long distances to complete the six steps … you start by submitting an application at the local street authority then to the Ward, thereafter to the Municipal, afterwards to the Regional Committee, which then passes the application to the Regional T&CM Secretariat and finally to the T&CM national council” *(Participant 4).
Barriers and challenges of the regulatory process	Capacity, knowledge, interest, and communication	*“Imagine a person appointed to regulate something that the practitioner is more knowledgeable on than the regulator … with less time to make regular supervision … no transport to facilitate visits … no meetings between them and practitioners…busy schedules with many assigned activities … once the practitioners know that they are rarely inspected they take this opportunity to commit all kinds of malpractices” *(Participant 21).

Abbreviation: T&CM, traditional and complementary medicine.

###  Awareness of T&CAM Regulatory Framework

 Several issues emerged with respect to awareness of T&CM regulatory framework in the country. The issues included awareness of and attitude towards existence of regulatory bodies and regulations; awareness of regulatory instruments and enforcements; supervision, inspection and information sharing; and regulators’ perspective on supervision and sharing of information. These issues are presented below.

####  Awareness of and Attitude Towards Existence of Regulatory Bodies and Regulations

 For an effective regulation process, there has to be a legally established functioning regulatory body, which is also legally known and recognized by the practitioners. Surprisingly, despite of the existence of a legal regulatory authority which is decentralized to all districts in the country, some respondents were not aware of such authorities, as it was confirmed by one practitioner.

*“I don’t know which body regulates me” *(Participant 9).

*“I have been practicing traditional/ alternative medicine for twelve years I have heard nothing called regulations. I wonder who set them and who was involved in developing them. Do they think this is conventional medicine?” *(Participant 2).

 However, majority of practitioners reported to be aware of the existence of laws, rules and regulations that guide T&CM operations.

 Additionally, those aware of the regulations were asked as to why the subsector is regulated. Reasons mentioned were: to protect traditional medicine consumers from possible abuse; to ensure that practitioners comply with the health provision standards; to regulate quality of products; to ensure safety of products; to regulate medicinal contents of the products; and to prevent false claims of treatment efficacy of traditional medicinal products. Nonetheless, some of those who reported to be aware of existence of the regulations, laws, and rules, did not know reasons for T&CM regulation.

 A few practitioners were totally against being regulated; they claimed that T&CM is an inherited practice that differs from one culture to another, and there is no common ground of operation to be regulated. They further argued that they had no formal training on T&CM practice, and therefore should not be regulated like the conventional medical practice.

*“I inherited this practice from our parents and grandparents…our practice is also rooted in our culture and traditions. Are these so-called regulators aware of our culture and traditions?” *(Participant 7).

 Another practitioner further argued:

*“This subsector is not supposed to be regulated […], our practice is inherited with varied conventions, which differ amongst cultures and has no common ground of practice, […], regulations should be for conventional medicine that is taught in school” *(Participant 16).

 Some practitioners characterized the T&CM practice as *“self-regulated.”* They further argued, a practice that emanates from inheritance has its own traditional self-regulating principles – roots and taboos – which cannot be regulated under modern practice rules and regulations.

 According to the Traditional Medicine Act of 2001 in Tanzania, T&CM practice and products must be licensed. Our results revealed that practitioners were not contented with the registration and licensing process. All interviewed practitioners branded the procedure as cumbersome, bureaucratic, and too costly. The practitioners complained that they had to complete 6 quite involving steps before they are registered and licensed to practice. One of the premises owners criticized the procedure and termed it as a barrier to development of the T&CM sub-sector in the country.

*“…it is a barrier to the practice…you have to travel long distances to complete the six steps….you start by submitting an application at the local street authority then to the Ward, thereafter to the Municipal, afterwards to the Regional Committee, which then passes the application to the Regional T*&*CM Secretariat and finally to the T*&*CM national council” *(Participant 4).

####  Awareness of Regulatory Instruments and Enforcements

 Availability of clearly understood regulatory instruments to both regulators and practitioners improves effectiveness of the regulatory process. The instruments include legislation, decrees, and guidelines. These instruments guide practice by spelling out what is required; translated by guidelines that guide implementation of the regulatory process. Only a few practitioners were able to mention most of the regulatory instruments used in the T&CM sub-sector. They commonly mentioned the Traditional Medicine Act 2001, the Traditional Medicine Board guidelines and the license and premises guidelines. Furthermore, some of the practitioners believed ignorance of the content of the regulatory instruments led to mushrooming of substandard and/or unlicensed premises, and poor practice. An experienced practitioner complained:

*“I know nothing about the instruments and their contents that regulators use to regulate our premises and practice” *(Participant 9).

 Furthermore, to improve effectiveness of the regulatory process, information on legal enforcement and penalties imposed for not adhering to the regulatory instruments must be communicated to practitioners. Awareness of such regulatory information might restrain malpractice. Most of the practitioners affirmed that penalties must be imposed to reduce unlawful practices. Although imposition of penalties was mentioned positively by many practitioners, only a few were aware of most of the respective penalties. The penalties mentioned by those few were: written warnings, temporary closure of premises, and revoking of license.

 Ignorance of the regulatory enforcements was openly revealed by some practitioners, one practitioner reported:

 “*I know nothing about penalties associated with breaching the laws and regulations in this business…and I do not know any penalty” *(Participant 6).

 Such ignorance of enforcement information could be used as a pretext by players in the market for malpractice. Therefore, participants in the T&CM market need to be informed of regulatory instruments and enforcements.

####  Supervision, Inspection, and Information Sharing 

 Apart from sharing enforcement information, regulators are supposed to share technical and operational information with practitioners during supervisory visits for the purpose of improving quality and safety of the practice. The regulatory framework (the Traditional Medicine Act and its guidelines) requires regulators to supervise and inspect T&CM premises, practice, and products regularly. Practitioners criticized the supervision and inspection process as weak and inefficient, with rare supervisory visits over the past year prior to this study. All practitioners interviewed could hardly remember the last time they were visited by a regulator. Some reported to have never seen a regulator visiting their premises ever since they started business. One practitioner confirmed:

 “*No regulator has ever visited these premises ever since I started practicing traditional medicine for the past twelve years now” *(Participant 3).

 These irregularities of supervision and inspection were blamed as the source of mushrooming of premises and practitioners that do not abide by regulations; because they know they are not inspected.

 To achieve regulatory effectiveness, regulators must communicate and share information with practitioners regularly. More than three quarters of the practitioners complained that information sharing with regulators was rarely happening. Practitioners believed information sharing should happen during inspection visits and since regulators rarely inspect the premises and practice, regulators were not performing as expected and hence perpetuating persistence of ignorance in T&CM practice. One practitioner confirmed:

*“Most of us practice with the little knowledge we have…. they shared information only once when I was registering long time ago… we have employees who are not aware of the regulatory information” *(Participant 10).

 Moreover, practitioners also reported that there were no open communication channels, apart from supervision, that would facilitate sharing of information. The missing channels mentioned included open discussion meetings, workshops, seminars, and brochures.

####  Regulators’ Perspective on Supervision and Sharing of Information

 The Tanzanian Traditional Medicine Act and its guidelines emphasize supportive supervision to be conducted by regulators to share information between the two sides. Nonetheless, regulators at the Municipal level apologetically reported to have made very few visits to premises in the past year prior to this study. Consequently, they had hardly shared T&CM information with practitioners. Five main reasons given for lack of effective supervision and information sharing were: multiple competing responsibilities (workload), tight working schedule, lack of resources including shortage of staff, lack of training, and poor knowledge on T&CM.

 Regarding competing responsibilities, the regulators complained that their superiors did not regard the T&CM regulatory task as primary, since they were not primarily employed as T&CM regulators. Having poor knowledge and lack of training on T&CM products and practice were highly underscored by regulators as conditions for ineffective supervision and sharing of information. All regulators were of an opinion that T&CM regulators should be people trained in the field and appointed to solely work as T&CM regulators. Lack of training was mentioned as the main impediment for sharing information with practitioners. All regulators at the Municipal confirmed to have not been trained on T&CM, as one of them complained:

*“Although I am a pharmacist … I have very scanty knowledge on traditional and alternative medicine. I cannot claim to be competent in supportive supervision” *(Participant 20).

 The Municipal regulators did not only candidly report their ignorance of T&CM, but also confirmed to have hardly shared information with practitioners:

*“…because I have poor knowledge in this field… I am not sure if I can confidently share such information with practitioners … they may be more informed than me” *(Participant 18).

 Regulators further reported that poor knowledge on T&CM affected their interest in performing regulatory duties. One regulator, a trained pharmacist, revealed that he does not have interest in traditional medicine because he is not sure of its efficacy and effectiveness in treating diseases:

*“… I am not even interested to be trained in traditional medicine … I do not believe in their efficacy … it will be difficult for me to work diligently and communicate effectively with the regulated” *(Participant 20).

 The negative attitude was further confirmed by practitioners as a cause of few and poor supervisory visits to premises and practitioners:

*“… you can easily identify inspectors with negative attitude … if he happens to visit your premises, he just concentrates on fault finding, nothing on supportive supervision” *(Participant 15).

 An official from the Ministry of Health reported to be aware of poor supervision made by regulators who have poor knowledge and negative attitude on traditional medicine:

*“…we have learned from practitioners that some local regulators do not implement supportive supervision … due to disinterest, poor knowledge and bad intentions … those with interest have good intentions and are supportive indeed, while those disinterested have bad intentions, they only look for mistakes and do not share information with practitioners for improvement” *(Participant 22).

 For improvement of the functions of the regulatory framework, 2 municipal regulators suggested that the hiring authority should employ and train people who have interest in the field; these people will be able to effectively conduct supportive supervision, communicate and share the right information, as well as coach and instruct practitioners to better abide by the regulations.

###  Adherence to Regulations 

 Practitioners mentioned several factors that influenced adherence to the regulatory process;

####  Financial Cost

 High financial cost associated with licensing and building or renting of standard business premises constrained adherence. Before a license is granted, the business premises must be in an acceptable condition. Other prohibitive costs included costly business license, costly product registration and diverse taxes levied by the municipal and income tax department.

*“One of the things that I remember in the regulatory guidelines is the structure and content of the premise, to adhere to this, it means a lot of money which most of us cannot afford to construct the premises the way it is needed by the guideline … it is very costly to register one herbal product, it costs around TZS 800 000 to TZS 1 000 000 (equivalent to $380 to $476) … cost of renting a standard premise as required by regulation one needs a lot of money”* (Participant 9).

####  Complexity of Laws and Regulations

 some practitioners complained of very stringent and tiresome rules, regulations, and guidelines, giving examples of the complex licensing and registration process.

*“…it is a barrier to the practice … you have to travel long distances to complete the six steps … you start by submitting an application at the local street authority then to the Ward, thereafter to the Municipal, afterwards to the Regional Committee, which then passes the application to the Regional T*&*CM Secretariat and finally to the T*&*CM national council” (*Participant 4).

###  Barriers and Challenges of the Regulatory Process 

 Both regulators and practitioners had an opinion that the regulatory authority was not as effective and efficient as it should be. Several challenges and impediments that contributed to poor functioning were mentioned.

####  Capacity, Knowledge, Interest, and Communication

 Challenges mentioned include low capacity and poor performance of both the T&CM national and local regulatory bodies. Other challenges mentioned were; poor knowledge of T&CM products and practice among regulators- majority of the regulators are converts of conventional medicine with very low knowledge on T&CM products, practice and policy; low interest in T&CM among regulators, hence there is less collaboration with practitioners; shortage of qualified staff at both national and municipal levels contribute to few supervisory visits; multiple competing responsibilities among regulators affecting their availability for supervision; unreliable means of transport causing untimely supervision; lack of regular and clear channels of communication between the regulators and practitioners; absence of on-job training on T&CM operations among regulators; frequent task shifting among local appointed regulators; and irregular supportive supervision visits to premises.

 The above challenges were reported by both practitioners and regulators to contribute to ineffectiveness of the regulatory process, facilitating malpractice and endangering consumer safety. One of the regulators summarized as follows:

*“Imagine a person appointed to regulate something that the practitioner is more knowledgeable on than the regulator … with less time to make regular supervision … no transport to facilitate visits … no meetings between them and practitioners … busy schedules with many assigned activities….once the practitioners know that they are rarely inspected they take this opportunity to commit all kinds of malpractices” *(Participant 21).

## Discussion

 The recommended criteria for assessing functioning and effectiveness of a regulatory framework suggests the inclusion of both regulators and practitioners in the assessment.^3^ The criteria, among others, include clarity, complexity and effectiveness of the regulations. This study explored the extent of imperfect information and challenges that the regulators and practitioners face during implementation of the T&CM regulatory process in Tanzania. Understandable and shared regulatory information is more likely to be adhered to by practitioners.^2^

 Imperfect information is one among many factors that might constrain awareness, and consequently, adherence to regulations. The situation could be more complicated when both the regulators and practitioners are not well informed on what is to be regulated and the corresponding regulations and guidelines. In this study, due to imperfect information, most practitioners were not aware of the regulations and guidelines governing the T&CM operations – they had rarely seen and/or read regulatory documents and/or met a regulator. Additionally, the municipal level regulators reported to have had scanty knowledge on T&CM products and operations. This situation may adversely affect health of customers due to poorly regulated practice and product standards in the T&CM industry in Tanzania.

 Worldwide, many people are visiting T&CM premises as their first point of treatment, and others increasingly consulting T&CM practitioners without consulting their doctors.^2,6,9,20^ This study found that the practitioners were less informed on the regulatory tools, requirements, and what was regulated. This gap of knowledge might be contributing to increasing malpractice – a negative effect of imperfect information.^21^ In Tanzania and other developing countries, T&CM practitioners don’t belong to organizations that are legally bound (with formal disciplinary codes, sanctions and procedures) to take legal actions against the practitioners’ malpractice.^20^ Absence of opportunities for the consumers to pursue complaints against practitioners’ malpractice, along with poor regulatory process, expose consumers health to risks. The T&CM practitioners being fully informed of the regulations might improve adherence to the regulatory process, and hence protect and promote the health of their consumers against risks.^5,16^

 For improvement of regulatory information and clinical practice, the literature suggests some solutions including, but not limited to, training on clinical standards and observation of professional practice, as well as creation of regulatory awareness by provision of education and information on laws and regulations to T&CM practitioners.^16^ Institutionally, it is suggested that promotion of awareness could be through a voluntary accreditation or self-regulation schemes and strengthening of the functioning regulatory authorities; initiation and strengthening of professional institutions for encouragement and promotion of professional standard practices among practitioners.^2,22,23^

 An effective regulatory process requires collaboration between the practitioners and regulators. A working collaboration is facilitated by acquisition of the necessary regulatory information by both the regulators and practitioners. In this study, heterogeneity in levels of knowledge about what should be regulated as well as the difference in what is practiced was revealed amongst practitioners. These differences could partly be influenced by the source and type of knowledge practiced. Some practitioners inherited – culturally rooted-practices and, some have experience without formal training and/or scientific training. This problem complicates the regulatory process due to cultural-rooted-practitioners’ failure to separate treatment from cultural heritage.^14^ Such heterogeneity of knowledge requires an effective training on both regulations and practice among practitioners. The training intervention would improve communication between regulators and practitioners, and consequently improve the regulatory results. The regulatory results would be weakened where practitioners have mixed knowledge on what should be practiced, which might affect communication between regulators and practitioners.

 The communication failure between regulators and practitioners is further aggravated by low knowledge on T&CM among regulators, as reported in this study. The lack of interest on T&CM among regulators together with the critical shortage of qualified human resources, increases the ineffectiveness of the regulatory process.^14,24^ Furthermore, Kayombo et al also revealed that implementation of traditional medicine rules, laws and regulations is still a problem in Tanzania, because most policy implementers are converts from conventional medical practice.^14^ Although the deployment of conventional medicine regulators is a way of integrating traditional medicine in the health system, those personnel who do not have formal training and interest in T&CM weaken the regulatory process, and act against the aim of integrating the subsector.^5^ This study reports that such regulators do not provide supportive supervision and cannot render the required and expected services to the subsector. Additionally, their scanty T&CM knowledge may lead to micromanagement of the practitioners, and consequently more malpractice.^5^

 Rare contact between regulators and practitioners caused low awareness and adherence to regulations. In general, this implies lack of partnership (involvement and sharing of information) in inspection and supervision, which might contribute to weakening of the regulatory process. It is argued that regulatory process would be effective and successful if communication and partnership between regulators and practitioners is fostered.^2^ Public private partnership could facilitate the effectiveness of the regulatory process by having a collaborated joint regulatory process that involves both regulators (public) and practitioners (private).^5^

 In line with literature, this study has identified challenges that also hamper the functioning of the T&CM regulatory authority, and consequently the regulatory process.^5^ Adherence to regulations is affected by costs associated with abiding by regulatory process. Respondents in this study revealed that license and registration fees, premises and other costs were too high, contributing to non-adherence of regulations among some practitioners. This high cost may contribute to abuse of the regulatory process through corruption and malpractice. Supply side challenges include those related to few human resources; low technical skills; lack of transport; and communication failure. These challenges affect the regulatory process (capacity, effectiveness and efficiency), assessment of safety and efficacy, and quality control in T&CM practice.^2,24^

###  Study Limitations

 Poor awareness of and adherence to T&CM regulations reported in this study should be interpreted with caution. The study was conducted in only one of the many regions of Tanzania, although it is where most of the T&CM registered premises are located. The study included only registered practitioners while there are other practitioners who are not registered who might have different opinions on the regulatory process. Studies are needed among registered and unregistered practitioners, to find out the extent to which the T&CM scientific knowledge is associated with understanding of and adherence to regulations, for establishing modalities of training practitioners on regulations and practicing standards to reduce malpractice and health risks. Furthermore, the challenges reported in this study ranged from socio-cultural-economic to technical, an examination on how investments in social and behavioral change interventions for practitioners, and use of technology for regulators is needed to find out how such investments might improve the T&CM regulatory process in Tanzania. Lastly, this study has targeted regulators and practitioners as the main implementers of regulatory framework, it would be informative to consider perspectives of end-users of T&CM as well to get insights on the outcome of inadequate T&CM knowledge among practitioners and regulators.

## Conclusion

 Existence of imperfect information among regulators and practitioners, as well as human resource, financial and infrastructural challenges affect effectiveness of T&CM regulatory process in Tanzania. Awareness of regulations among practitioners and presence of knowledgeable regulators would facilitate adherence to T&CM regulations and promote practice safety. For an effective T&CM regulation process, both regulators and practitioners must collaborate and share information regularly. Existence of imperfect information should be addressed by the regulatory authority by training both regulators and practitioners on T&CM practice, procedures, legislation, guidelines and regulations. Moreover, existing human resource, financial and infrastructural challenges facing both regulators and practitioners must be addressed to improve T&CM regulation adherence.

## Acknowledgements

 We would like to thank Sigwa Group for financial assistance in data collection, and respondents for being willing to respond to the questions. The funder had no role in conducting this study.

## Ethical issues

 The study was approved by the Muhimbili University of Health and Allied Sciences Institutional Review Board (ethical reference letter MU/PGD/SAEC/VOL.X/07/2017).

## Competing interests

 Authors declare that they have no competing interests.

## Authors’ contributions

 PGM: conception and design; acquisition of data; analysis and interpretation of data; drafting of the manuscript; critical revision of the manuscript for important intellectual content; administrative, technical, or material support; and supervision. HPS: analysis and interpretation of data; drafting of the manuscript; critical revision of the manuscript for important intellectual content; and administrative, technical, or material support.

## Funding

 Sigwa Group Tanzania funded data collection.
